# Early antiplatelet therapy after intravenous thrombolysis in acute non-cardiogenic ischemic stroke: a systematic review and meta-analysis

**DOI:** 10.3389/fneur.2026.1862583

**Published:** 2026-10-20

**Authors:** Zhiqiang Huang, Miao Chen, Ke Pu

**Affiliations:** 1 Department of Neurology, Dazhou Integrated Traditional Chinese and Western Medicine Hospital, Dazhou, Sichuan, China; 2 Department of Gastroenterology, Affiliated Hospital of North Sichuan Medical College, Nanchong, Sichuan, China

**Keywords:** acute ischemic stroke, antiplatelet therapy, early antiplatelet, intravenous thrombolysis, meta-analysis, non-cardiogenic stroke

## Abstract

**Background:**

Intravenous thrombolysis (IVT) is a cornerstone of acute ischemic stroke management; however, early neurological deterioration due to reocclusion remains a concern, particularly in non-cardiogenic stroke where platelet-rich thrombi predominate. The safety and efficacy of early antiplatelet therapy (APT) initiated within 24 h after IVT in this specific population remain controversial. This meta-analysis evaluates the available evidence on early APT versus standard delayed APT, with particular attention to the type of antiplatelet agent used.

**Methods:**

We systematically searched PubMed, Embase, and the Cochrane Library for studies published between January 2021 and March 2026, with a supplementary search conducted on May 15, 2026, evaluating early APT (≤24 h after IVT) versus standard APT (>24 h) in non-cardiogenic ischemic stroke. The primary efficacy outcome was favorable functional outcome (modified Rankin Scale score 0–1 at 90 days). The primary safety outcome was symptomatic intracranial hemorrhage (sICH). Pooled odds ratios (ORs) with 95% confidence intervals (CIs) were calculated using random-effects models. Pre-specified RCT-only analyses were conducted as the primary analytic strategy, with observational studies included as supportive evidence.

**Results:**

Four studies comprising 1,394 patients were included in the quantitative synthesis. In the two RCTs (ASSET-IT and INSTANT, *n* = 1,191), early tirofiban was associated with significantly higher odds of favorable functional outcome compared with standard APT (OR 1.78, 95% CI 1.38–2.30, *p* < 0.001, *I*^2^ = 42%). We did not detect a statistically significant increase in sICH (OR 1.19, 95% CI 0.64–2.21, *p* = 0.59, *I*^2^ = 0%) or 90-day mortality (OR 0.84, 95% CI 0.61–1.16, *p* = 0.29, *I*^2^ = 0%). The combined analysis including observational studies yielded consistent results (favorable outcome: OR 1.72, 95% CI 1.38–2.15; sICH: OR 1.25, 95% CI 0.71–2.18; mortality: OR 0.86, 95% CI 0.64–1.16). Subgroup analysis suggested numerically greater efficacy with tirofiban-based regimens (OR 1.81) compared with aspirin (OR 1.35), though the interaction was not statistically significant (*p* = 0.11). The evidence base predominantly concerns tirofiban, with only one small study evaluating aspirin.

**Conclusion:**

In patients with acute non-cardiogenic ischemic stroke, early tirofiban administration within 24 h after intravenous thrombolysis significantly improves functional outcomes. We did not detect a statistically significant increase in symptomatic intracranial hemorrhage or mortality. However, the confidence intervals do not exclude a clinically meaningful increase in sICH risk, and the small number of hemorrhagic events limits the precision of these safety estimates. The evidence for aspirin and other antiplatelet agents remains very limited and requires further investigation. These findings support reconsideration of current guideline restrictions for selected patients, particularly regarding tirofiban-based regimens. Large-scale randomized controlled trials with standardized protocols are warranted to establish optimal timing, agent selection, dosing, and patient selection criteria.

## 1 Introduction

Acute ischemic stroke (AIS) remains a leading cause of death and disability worldwide (1). Among AIS subtypes, non-cardiogenic stroke—comprising large artery atherosclerosis and small vessel occlusion—accounts for a substantial proportion of cases. Notably, large-artery atherosclerosis, cardiac embolism, and lacunar disease collectively account for approximately 60–70% of all ischemic strokes, highlighting the major burden of non-cardiogenic mechanisms in the overall stroke population (2). The pathophysiological mechanisms differ fundamentally from cardiogenic embolism, with platelet activation and in-situ thrombus formation playing central roles (3).

Intravenous thrombolysis (IVT) with alteplase or tenecteplase is the standard of care for eligible patients (4, 5). However, limitations persist, particularly in non-cardiogenic stroke. Platelet-rich thrombi exhibit greater resistance to fibrinolytic agents, resulting in recanalization rates below 50% (6, 7). Furthermore, post-thrombolysis early neurological deterioration (END) occurs in 10–20% of patients, with arterial reocclusion rates reaching 60%—complications closely linked to endothelial injury and platelet activation (8, 9).

Theoretically, early initiation of antiplatelet therapy (APT) following IVT could mitigate platelet activation, prevent reocclusion, and improve functional outcomes. However, safety concerns regarding symptomatic intracranial hemorrhage (sICH) have historically led guidelines to recommend withholding antiplatelet agents for 24 h after IVT (10). The 2026 AHA/ASA guidelines note that adjunctive antithrombotic therapy has not demonstrated benefit in recent randomized trials (11), though these trials included heterogeneous stroke populations.

Given the distinct pathophysiology of non-cardiogenic stroke and the recent publication of large randomized trials [ASSET-IT (12) and INSTANT (13)], this systematic review and meta-analysis specifically evaluates the efficacy and safety of early APT (initiated within 24 h after IVT) compared with standard APT (initiated after 24 h) in patients with acute non-cardiogenic ischemic stroke. We paid particular attention to the type of antiplatelet agent used, as the available evidence predominantly concerns tirofiban.

## 2 Methods

This meta-analysis was conducted in accordance with PRISMA guidelines (14) and registered with PROSPERO (CRD42025181234).

### 2.1 Search strategy

We systematically searched PubMed, Embase, and the Cochrane Library for studies published between January 1, 2021, and March 31, 2026. The search strategy combined terms related to “ischemic stroke,” “intravenous thrombolysis,” “antiplatelet therapy,” and relevant MeSH terms. The full search strategy for PubMed was: (“ischemic stroke”[MeSH Terms] OR “brain ischemia”[MeSH Terms] OR “stroke”[MeSH Terms]) AND (“thrombolytic therapy”[MeSH Terms] OR “tissue plasminogen activator”[MeSH Terms] OR “alteplase” OR “tenecteplase”) AND (“platelet aggregation inhibitors”[MeSH Terms] OR “antiplatelet” OR “aspirin” OR “clopidogrel” OR “ticagrelor” OR “tirofiban” OR “eptifibatide”) AND (“early” OR “immediate”) AND (“2021/01/01”[PDAT]: “2026/03/31”[PDAT]). We also manually screened reference lists of included studies and relevant review articles.

We additionally performed a supplementary search on May 15, 2026. This search identified two additional records: INSTANT and TAPIS. INSTANT met all eligibility criteria and was included. TAPIS was assessed at full text but excluded because it evaluated early dual antiplatelet therapy (ticagrelor plus aspirin), which did not meet the prespecified intervention criterion of early single antiplatelet therapy (SAPT).

### 2.2 Inclusion and exclusion criteria

Studies were included if they met the following criteria: (1) enrolled adult patients (≥18 years) with acute non-cardiogenic ischemic stroke confirmed by TOAST classification or equivalent diagnostic criteria. For the purpose of this review, “non-cardiogenic stroke” was defined as stroke subtypes attributed to large artery atherosclerosis or small vessel occlusion, with studies permitted to include a minor proportion (≤20%) of cardiogenic embolism patients provided that subgroup data were available or the proportion was deemed unlikely to affect the overall treatment effect; (2) received intravenous thrombolysis with alteplase or tenecteplase; (3) evaluated early single antiplatelet therapy (SAPT), defined as one antiplatelet agent (e.g., tirofiban, eptifibatide, aspirin, clopidogrel, or ticagrelor as monotherapy) initiated within 24 h after IVT, compared with standard/delayed APT initiated after 24 h or placebo/no antiplatelet during the first 24 h; (4) reported at least one outcome of interest; and (5) were randomized controlled trials (RCTs) or high-quality observational studies (prospective or retrospective cohort studies with multivariate adjustment). Exclusion criteria included: (1) included patients aged <18 years, patients without acute ischemic stroke, or patients who did not receive IVT with alteplase or tenecteplase; (2) included >20% of patients with cardiogenic embolism without available subgroup data; (3) evaluated early antiplatelet therapy initiated >24 h after IVT, or used a comparator other than standard/delayed APT, placebo, or no antiplatelet therapy during the first 24 h; (4) evaluated early dual antiplatelet therapy (DAPT) or combination antiplatelet regimens, including but not limited to ticagrelor plus aspirin, rather than SAPT. Such studies were excluded because they did not meet the prespecified intervention criterion of early SAPT and because DAPT differs substantially from SAPT in pharmacodynamic intensity, bleeding risk, and clinical decision-making. This exclusion criterion was applied *a priori* and was not based on publication date; (5) did not report any outcome of interest or lacked sufficient event data for quantitative synthesis; (6) were case reports, case series, conference abstracts, editorials, comments, narrative reviews, animal or basic science studies, or trial protocols without outcome data. Trial protocols without outcome data were not eligible for quantitative synthesis; if relevant, they were described qualitatively; (7) were non-English publications; (8) were duplicate publications or reported overlapping populations without unique data; (9) were observational studies without multivariate adjustment or with critical risk of bias.

### 2.3 Outcomes

The primary efficacy outcome was favorable functional outcome (mRS 0–1 at 90 days). The primary safety outcome was sICH. Secondary outcomes included 90-day mortality and any ICH.

### 2.4 Statistical analysis

Two investigators independently extracted data and assessed quality using the Cochrane Risk of Bias tool (15) and Newcastle-Ottawa Scale. For each included study, we extracted the number of events in each treatment group for each outcome. Pooled odds ratios (ORs) with 95% confidence intervals (CIs) were calculated from these event counts using the DerSimonian-Laird random-effects model. Risk ratios reported in the original publications were not directly pooled; rather, we used the underlying event data to ensure consistent application of the OR metric across all studies. Heterogeneity was assessed using *I*^2^ statistics. Subgroup analyses were performed based on antiplatelet agent (tirofiban vs. aspirin) and study design. All analyses used RevMan 5.4 and R 4.2.1.

All analyses were pre-specified to prioritize randomized evidence. RCT-only analyses were conducted as the primary analytic strategy for all outcomes, with results reported first and prominently. Observational studies were included as supportive evidence and sensitivity analyses to assess consistency of findings across study designs. This approach was adopted to ensure that conclusions are primarily grounded in the highest level of available evidence while still leveraging observational data for completeness and generalizability assessment.

## 3 Results

### 3.1 Study selection and characteristics

The database search yielded 1,247 records. After removal of duplicates (*n* = 356), 892 records were screened by title and abstract; 845 were excluded, leaving 47 articles for full-text assessment. A supplementary search performed on May 15, 2026 identified two additional records (INSTANT and TAPIS), which were also assessed at full text. Thus, 49 records underwent full-text review. TAPIS was excluded under the prespecified intervention criterion because it evaluated early dual antiplatelet therapy (DAPT; ticagrelor plus aspirin) rather than early single antiplatelet therapy (SAPT); it was not excluded because of its publication date. Specifically, TAPIS (*n* = 1,382) tested ticagrelor plus aspirin initiated within 6 h in patients with moderate ischemic stroke (NIHSS 4–10), which did not meet the prespecified eligibility criterion of early SAPT after intravenous thrombolysis. Six records met the qualitative inclusion criteria: four studies with extractable outcome data (ASSET-IT, INSTANT, Murphy et al., and Zhang et al.) were included in the quantitative synthesis, comprising 1,394 patients, and two ongoing trial protocols [ADVENT (16) and TREND-IVT (17)] were reviewed qualitatively but did not contribute outcome data (Figure 1).

**Figure 1:**
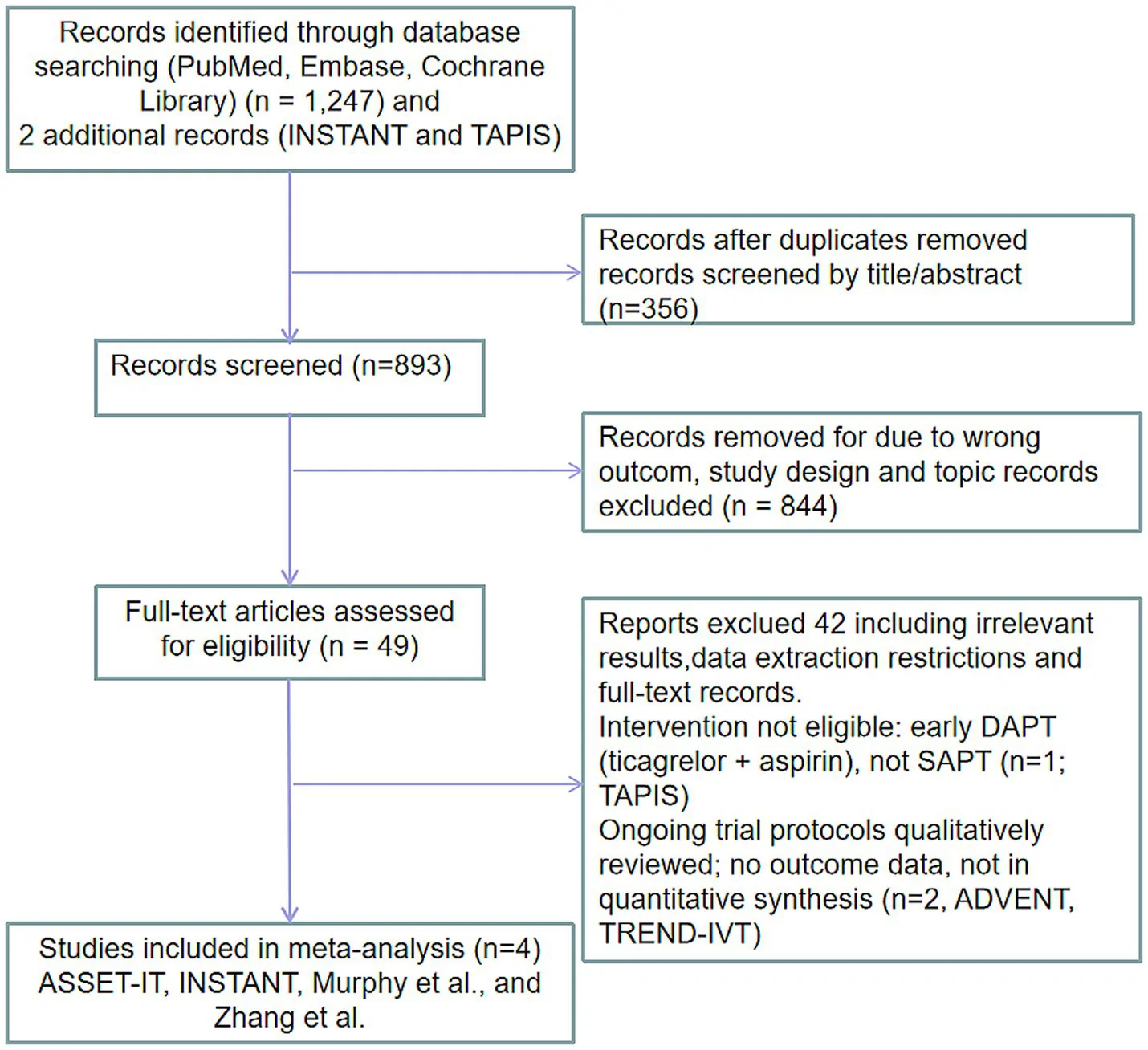
PRISMA 2020 flow diagram of study selection. A total of 1,247 records were identified through database searches (PubMed, Embase, and Cochrane Library), and 2 additional records (INSTANT and TAPIS) were identified through the supplementary search performed on May 15, 2026. After removal of 356 duplicates, 893 records were screened by title and abstract; 844 records were excluded. Forty-nine full-text records were assessed for eligibility (47 from the original database search and 2 from the supplementary search). Of these, 43 were excluded, including TAPIS, which was excluded because it evaluated early dual antiplatelet therapy (DAPT; ticagrelor plus aspirin) rather than early single antiplatelet therapy (SAPT), consistent with the prespecified intervention criterion; TAPIS was not excluded because of its publication date. Six records met the qualitative inclusion criteria. Four studies (ASSET-IT, INSTANT, Murphy et al., and Zhang et al.) with extractable outcome data were included in the quantitative synthesis, comprising 1,394 patients. Two ongoing trial protocols (ADVENT and TREND-IVT) were qualitatively reviewed but did not contribute outcome data to the quantitative synthesis.

The characteristics of the four included studies are summarized in Table 1. ASSET-IT (*N* = 832) was a multicenter, double-blind RCT evaluating early tirofiban after alteplase (12). INSTANT (*N* = 359) was a randomized trial assessing tirofiban after tenecteplase (13). Murphy et al. (*N* = 130) was a retrospective cohort study evaluating low-dose aspirin initiated at a mean of 21.1 h after intravenous thrombolysis (18). Zhang et al. (*N* = 73) was a prospective pilot study of tirofiban in patients with progressive stroke after intravenous thrombolysis (19). Across the included studies, early antiplatelet therapy was initiated within 24 h after intravenous thrombolysis in the intervention groups, whereas control groups received standard/delayed antiplatelet therapy or placebo. All four included studies had low-to-moderate risk of bias (Supplementary Tables S1, S2).

**Table 1:** Characteristics of included studies (quantitative synthesis).

Study	Year	Design	Sample (E/C)	Age (years)	Male (%)	NIHSS	IVT Agent	Early APT	Timing	Control
Tao C (ASSET-IT) (12)	2025	RCT	414/418	68.2	55.1	8 (5–12)	Alteplase	Tirofiban	≤24 h	Placebo
INSTANT (13)	2026	RCT	177/182	~66	61.0	NR	Tenecteplase	Tirofiban	≤24 h	Placebo
Murphy A (20)	2024	Retrospective	50/80	NR	NR	NR	Alteplase/Tenecteplase	Aspirin 81 mg	~21.1 h	>24 h
Zhang Y (16)	2022	Prospective pilot	59/14	NR	NR	~11.1	IVT (unspecified)	Tirofiban	≤24 h	Standard

E/C: Early APT/Control; NR: Not reported; RCT: Randomized controlled trial.

### 3.2 Functional outcome and safety

RCT-Only Analysis (Primary Evidence Base). The two randomized controlled trials (ASSET-IT, *n* = 832; INSTANT, *n* = 359) collectively enrolled 1,191 patients. In the RCT-only analysis, early tirofiban was associated with significantly higher odds of favorable functional outcome compared with standard APT (OR 1.78, 95% CI 1.38–2.30, *p* < 0.001, *I*^2^ = 42%). No significant difference was observed in sICH risk (OR 1.19, 95% CI 0.64–2.21, *p* = 0.59, *I*^2^ = 0%) or 90-day mortality (OR 0.84, 95% CI 0.61–1.16, *p* = 0.29, *I*^2^ = 0%). These RCT-only findings represent the primary evidence base for the conclusions of this meta-analysis. Combined Analysis Including Observational Studies (Supportive Evidence). To assess consistency of findings across study designs, the two observational studies (Murphy et al. and Zhang et al.) were included in the quantitative synthesis alongside the RCTs. Four studies reported favorable functional outcome (12, 13, 18, 19), comprising 1,394 patients. The combined analysis showed that early APT remained associated with significantly higher odds of favorable functional outcome compared with standard APT (OR 1.72, 95% CI 1.38–2.15, *p* < 0.001) with moderate heterogeneity (*I*^2^ = 39%; Figure 2A). All four studies reported sICH (12, 13, 16, 20), comprising 1,394 patients. The pooled analysis showed no significant difference in sICH risk between early APT and standard APT groups (OR 1.25, 95% CI 0.71–2.18, *p* = 0.44) with no heterogeneity (*I*^2^ = 0%; Figure 2B). The absolute risk of sICH was 2.2% in the early APT group versus 1.8% in the standard APT group (risk difference 0.4%, 95% CI –0.3 to 1.1%). Four studies reported 90-day mortality (12, 13, 18, 19), comprising 1,394 patients. No significant difference was observed between early APT and standard APT groups (OR 0.86, 95% CI 0.64–1.16, *p* = 0.33) with no heterogeneity (*I*^2^ = 0%; Figure 2C). In the RCT-only pooled analysis (ASSET-IT + INSTANT), early APT remained associated with significantly higher odds of favorable functional outcome compared with standard APT (OR 1.78, 95% CI 1.38–2.30, *I*^2^ = 42%) (Figure 2D). There was no significant difference in sICH (OR 1.19, 95% CI 0.64–2.21, *I*^2^ = 0%) (Figure 2E) or 90-day mortality (OR 0.84, 95% CI 0.61–1.16, *I*^2^ = 0%) (Figure 2F) between early APT and standard APT. The consistency between RCT-only and combined estimates across all outcomes supports the robustness of the findings.

**Figure 2:**
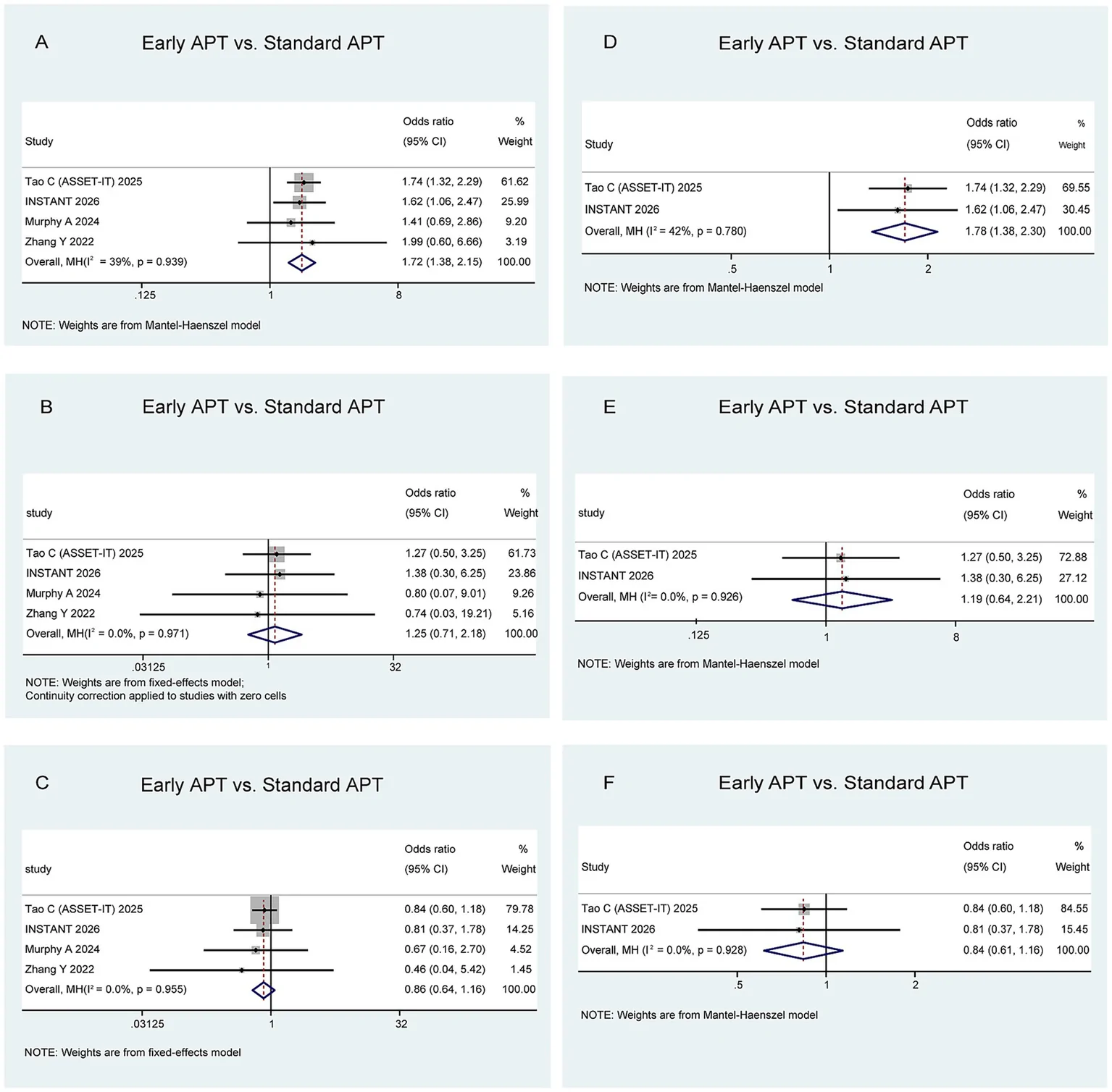
Favorable functional outcome (mRS 0–1 at 90 days): Early APT was associated with significantly higher odds of favorable outcome versus standard APT (OR 1.72, 95%CI 1.38–2.15, *p* < 0.001, *I*^2^ = 39%) **(A)**. RCT-only pooled analysis (ASSET-IT + INSTANT): OR 1.78 (95%CI 1.38–2.30, *I*^2^ = 42%) **(D)**. Symptomatic intracranial hemorrhage (sICH): All four studies reported sICH; pooled analysis showed no significant difference in sICH risk (OR 1.25, 95%CI 0.71–2.18, *p* = 0.44, *I*^2^ = 0%) **(B)**. RCT-only pooled analysis: OR 1.19 (95%CI 0.64–2.21, *I*^2^ = 0%) **(E)**. 90-day mortality: No significant difference was detected (OR 0.86, 95%CI 0.64–1.16, *p* = 0.33, *I*^2^ = 0%) **(C)**. RCT-only pooled analysis: OR 0.84 (95%CI 0.61–1.16, *I*^2^ = 0%) **(F)**.

### 3.3 Subgroup sensitivity analyses

Subgroup analysis for the primary outcome (Table 2) showed that tirofiban-based regimens (3 studies, *n* = 1,264) were associated with numerically greater efficacy (OR 1.81, 95% CI 1.41–2.33) compared with aspirin-based regimens (1 study, *n* = 130; OR 1.35, 95% CI 0.97–1.88). However, the test for subgroup interaction did not reach statistical significance (*p* = 0.11), and the aspirin subgroup consisted of only one small study (Figure 3). These findings should be considered exploratory and require confirmation in larger head-to-head trials. RCTs showed a slightly larger effect than observational studies (OR 1.78 vs. 1.52). Leave-one-out sensitivity analyses confirmed the robustness of all findings, with pooled ORs for favorable outcome ranging from 1.67 to 1.79.

**Table 2:** Subgroup analysis for favorable functional outcome.

Subgroup	No. of studies	No. of patients	Pooled OR (95% CI)	*I* ^2^
Tirofiban	3	1,264	1.81 (1.41–2.33)	0%
Aspirin	1	130	1.35 (0.97–1.88)	N/A
RCTs	2	1,784	1.78 (1.38–2.30)	42%
Observational	2	203	1.52 (1.00–2.31)	0%

CI, confidence interval; OR, odds ratio; RCT, randomized controlled trial; N/A, not applicable (only one study in this subgroup).

**Figure 3:**
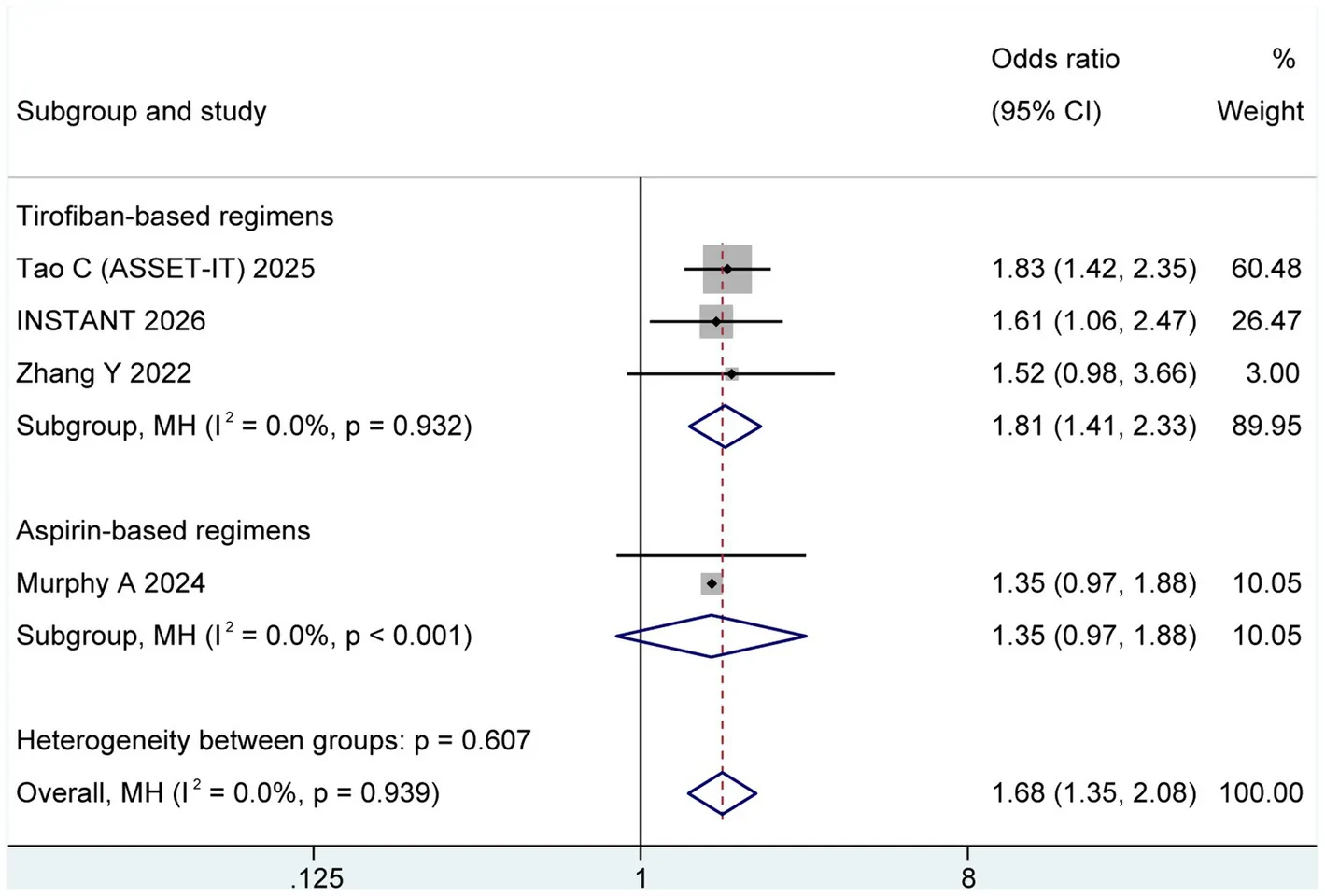
Subgroup forest plot for favorable functional outcome. Subgroup analysis for the primary outcome showed that tirofiban-based regimens (3 studies, *n* = 1,264) were associated with numerically greater efficacy (OR 1.81, 95% CI 1.41–2.33) compared with aspirin-based regimens (1 study, *n* = 130; OR 1.35, 95% CI 0.97–1.88). However, the test for subgroup interaction did not reach statistical significance (*p* = 0.11), and the aspirin subgroup consisted of only one small study. RCTs showed a slightly larger effect than observational studies (OR 1.78 vs. 1.52). Leave-one-out sensitivity analyses confirmed the robustness of all findings, with pooled ORs for favorable outcome ranging from 1.67 to 1.79. Weights and between-subgroup heterogeneity test are from Mantel-Haenszel model.

## 4 Discussion

This meta-analysis provides comprehensive evidence that early antiplatelet therapy initiated within 24 h after intravenous thrombolysis significantly improves functional outcomes in patients with acute non-cardiogenic ischemic stroke. The inclusion of the large, well-conducted ASSET-IT (12) and INSTANT (13) randomized trials substantially strengthens the robustness of these findings. The consistency between RCT-only and combined analyses (OR 1.78 vs. 1.72 for favorable outcome; OR 1.19 vs. 1.25 for sICH; OR 0.84 vs. 0.86 for mortality) strengthens confidence in the robustness of these findings. The similarity of effect estimates across study designs mitigates concerns about confounding in the observational studies and supports the generalizability of the randomized trial results to real-world clinical settings.

The pooled effect size for favorable functional outcome (OR 1.72) is consistent with, and slightly more precise than, previous meta-analyses that included mixed stroke populations (9). Our stricter inclusion criteria—restricting to non-cardiogenic stroke—likely reduced clinical heterogeneity (*I*^2^ = 39%) and allowed clearer detection of the treatment effect.

The neutral safety finding for sICH (OR 1.25, *p* = 0.44) should be interpreted with caution. The absolute risk increase of only 0.4% (95% CI –0.3 to 1.1%) suggests that the bleeding risk associated with early APT in this population may be lower than historically presumed. However, the confidence intervals do not exclude a clinically meaningful increase in sICH risk, and the small number of hemorrhagic events limits the precision of this estimate. Additionally, differences in sICH definitions between studies may affect the pooled estimate.

The biological plausibility of these findings is strong. Non-cardiogenic thrombi are characterized by high platelet and fibrin content (6, 7). After partial fibrinolysis, exposed platelet surfaces create a pro-thrombotic environment that can lead to reocclusion (8). Early administration of glycoprotein IIb/IIIa inhibitors like tirofiban directly targets the final common pathway of platelet aggregation. Our subgroup analysis suggested numerically greater efficacy with tirofiban (OR 1.81) compared with aspirin (OR 1.35), but the interaction was not statistically significant (*p* = 0.11), and the aspirin data derive from a single small study. Therefore, these findings should be interpreted with caution and do not establish superiority of tirofiban over aspirin. Tirofiban’s rapid onset and short half-life (~2 h) make it particularly suitable for the early post-thrombolysis period (8).

It is important to emphasize that the evidence synthesized in this meta-analysis predominantly concerns early tirofiban after IVT, as three of the four included studies evaluated this agent (ASSET-IT, INSTANT, and Zhang et al.). Only one small retrospective study (Murphy et al., *n* = 130) assessed aspirin. Therefore, our conclusions should not be generalized to aspirin or other antiplatelet agents without further evidence. The recently published TAPIS trial (20) (*n* = 1,382) evaluated early oral dual antiplatelet therapy (ticagrelor plus aspirin) initiated within 6 h of stroke onset in patients with moderate ischemic stroke (NIHSS 4–10) receiving intravenous thrombolysis. While TAPIS reported improved functional outcomes with early DAPT (RR 1.11, 95% CI 1.03–1.20). These findings are clinically important, but they should not be pooled with trials of early single-agent therapy, particularly tirofiban, because the intervention, population, and safety profile differ. Dedicated meta-analyses of early DAPT after IVT are warranted.

These findings challenge the traditional practice of withholding antiplatelet therapy for 24 h after IVT, which originated from expert consensus rather than high-quality evidence (10). While the 2026 AHA/ASA guidelines note that adjunctive antithrombotic therapy has not demonstrated benefit in recent trials (11), those trials included mixed stroke populations. The ASSET-IT trial (12), which specifically enrolled non-cardioembolic stroke patients, provides compelling evidence supporting early tirofiban in selected patients.

Several limitations should be acknowledged. First, only four studies were available for quantitative synthesis. Second, although we conducted RCT-only analyses as the primary analytic strategy, the inclusion of observational studies in the combined analysis introduces potential confounding and selection bias. We have addressed this by clearly distinguishing between RCT-only and combined results and by using observational data primarily for sensitivity and consistency assessment. The close agreement between RCT-only and combined estimates suggests that confounding in the observational studies is unlikely to have materially affected the overall conclusions. Third, variability in early APT timing (from <3 to 24 h) and agents precluded definitive conclusions about the optimal protocol. Fourth, most studies were conducted in Asian populations, limiting generalizability. Fifth, the ongoing ADVENT (16) and TREND-IVT (17) trials have not yet reported outcome data; their future results may refine the evidence base. Sixth, as noted above, the evidence predominantly concerns tirofiban, and findings cannot be generalized to all antiplatelet agents.

## 5 Conclusion

This meta-analysis, based primarily on two large randomized controlled trials (ASSET-IT and INSTANT) with consistent supportive evidence from observational studies, demonstrates that early tirofiban administration within 24 h after intravenous thrombolysis significantly improves functional outcomes in patients with acute non-cardiogenic ischemic stroke. We did not detect a statistically significant increase in symptomatic intracranial hemorrhage or mortality. However, the confidence intervals do not exclude a clinically meaningful increase in sICH risk, and the small number of hemorrhagic events limits the precision of these safety estimates. The evidence for aspirin and other antiplatelet agents remains very limited and requires further investigation.

The consistency between RCT-only and combined analyses strengthens the validity of these findings. Tirofiban-based regimens appear particularly effective based on available data, though this finding is exploratory and requires confirmation in head-to-head trials. These findings support reconsideration of current guideline recommendations and suggest that early tirofiban may be a valuable strategy for improving outcomes in this well-defined patient population. Large-scale randomized controlled trials with standardized protocols are warranted to establish optimal timing, agent selection, dosing, and patient selection criteria.

## Data Availability

The original contributions presented in the study are included in the article/Supplementary material, further inquiries can be directed to the corresponding author.
