# Supplementary material for: Early antiplatelet therapy after intravenous thrombolysis in acute non-cardiogenic ischemic stroke: a systematic review and meta-analysis
Source: Front Neurol. 2026 Oct 20;17:1862583. doi: 10.3389/fneur.2026.1862583 (PMC13727430; doi:10.3389/fneur.2026.1862583)
Supplement: Supplementary file 1 [file Table_1.DOCX]

Supplementary Material

# 1 Supplementary Tables

**Supplementary Table S1. Risk of Bias Assessment for Randomized Controlled Trials (Cochrane RoB 2.0 Tool)**

| Study | Domain 1: Randomization process | Domain 2: Deviations from intended interventions | Domain 3: Missing outcome data | Domain 4: Measurement of outcome | Domain 5: Selection of reported results | Overall Risk of Bias |
| --- | --- | --- | --- | --- | --- | --- |
| Tao C (ASSET-IT) 2025 | Low | Low | Low | Low | Low | **Low** |
| INSTANT 2026 | Low | Low | Low | Low | Low | **Low** |
| Zhang Y 2022 | Some¹ | Low | Low | Low | Low | **Some** |

¹Open-label design without sham infusion; outcome assessors were blinded.

**Supplementary Table S2. Quality Assessment for Observational Studies (NOS, 9-star scale)**

| Study | Selection (max 4★) | Comparability (max 2★) | Outcome (max 3★) | **Total** |
| --- | --- | --- | --- | --- |
| Murphy A 2024 | ★★★★ | ★★ | ★★★ | **9/9** |
| Zhang Y 2022¹ | ★★★★ | ★ | ★★★ | **8/9** |

¹Limited multivariate adjustment; no adjustment for door-to-needle time or baseline glucose.
